# Pediatric Emergency Department Responses to COVID-19: Transitioning From Surge Preparation to Regional Support

**DOI:** 10.1017/dmp.2020.197

**Published:** 2020-06-18

**Authors:** Emily A. Hartford, Ashley Keilman, Hiromi Yoshida, Russell Migita, Todd Chang, Brianna Enriquez, Deborah R. Liu

**Affiliations:** Pediatric Emergency Medicine, Seattle Children’s Hospital, Seattle, WA; Pediatric Emergency Medicine, Children’s Hospital of Los Angeles, Los Angeles, CA

**Keywords:** emergency medicine, emergency preparedness, pandemics

## Abstract

In the midst of a global pandemic, hospitals around the world are working to meet the demand for patients ill with the 2019 coronavirus disease (COVID-19) caused by the novel coronavirus first identified in Wuhan, China. As the crisis unfolds, several countries have reported lower numbers as well as less morbidity and mortality for pediatric patients. Thus, pediatric centers find themselves pivoting from preparing for a patient surge to finding ways to support the regional response for adults. This study describes the response from 2 West Coast freestanding academic children’s hospitals that were among the first cities in the United States impacted during this pandemic.

The coronavirus disease (COVID-19) was first identified as a new virus in Wuhan, China, in December 2019. Over the next few months, it spread worldwide causing the World Health Organization to declare a pandemic on March 11, 2020.^1^ The first case in the United States was reported on January 21, 2020, just outside of Seattle, Washington.^2^ Since then, every state in the United States has reported cases of COVID-19, and most have closed schools, non-essential services, and instituted stay-at-home orders. As of April 13, the United States has the highest total number of reported COVID-19 cases of any country at over 580 000, with over 1.9 million cases reported around the world.^3^

As the world continues to learn about this novel human pathogen, 1 peculiar theme is the lower likelihood of severe disease in children. In an early review from China, it was reported that over 90% of children with COVID-19 had mild or moderate disease, 4.4% were asymptomatic, and very few presented with hypoxia (5.3%) or critical illness (0.6%).^4^ It remains unclear whether children are less likely to be infected with COVID-19, if they are infected and manifest less severe disease, or if there is a combined effect.^5^ Children have comprised a very small proportion of overall confirmed COVID-19 cases in both Italy and China, with only 1 confirmed death reported.^6,7^ In the United States, as of April 3, only 1.7% of all confirmed cases are children (< 18 years), and children are less likely than adults to have symptoms of fever, cough, and/or shortness of breath. Children are also less likely than adults to be hospitalized or to require intensive care. Thus far, in the United States, there have been 3 pediatric deaths reported and reviews are underway to determine whether the cause of these deaths was indeed COVID-19.^8^

As hospitals around the world prepare and respond to this crisis, pediatric centers have been affected to a lesser degree. We aim to describe the stages of pandemic response at 2 major academic freestanding children’s hospitals on the West Coast of the United States, to highlight the operational changes implemented and barriers encountered during the response, and to discuss the transition from preparing for a surge of pediatric patients to supporting regional hospitals as pediatric volumes remain low.

## OVERVIEW OF THE EMERGENCY DEPARTMENT RESPONSE FOR SEATTLE CHILDREN’S HOSPITAL (SCH) AND CHILDREN’S HOSPITAL OF LOS ANGELES (CHLA)

See Table 1 for an overview of the response for both institutions.

### SCH: Timeline

**FIGURE 1:**
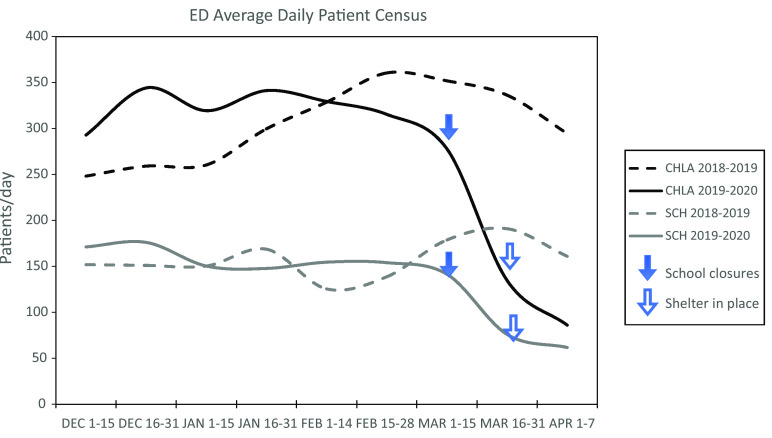
Averaged Daily Census in 2-week Blocks for CHLA ED and SCH ED During Winter 2018–2019 and Winter 2019–2020 (as COVID-19 Pandemic Unfolded).

On January 21, the first known case of COVID-19 in the United States was reported near Seattle, Washington, in an individual returning from mainland China. He was identified and placed in quarantine. On February 7, SCH saw our first person under investigation (PUI) tested for COVID-19 by the Washington State Department of Health (DOH). The initial volume of exposed patients was low and patients were discussed with DOH to determine testing eligibility. On February 28, we reported the second known COVID-19 case in Washington State in a teenager with no travel history, no known exposure and mild disease, marking the first identification of community transmission. The first death was reported on February 29 in a 52-year-old patient with underlying medical conditions who had visited a long-term care facility that was subsequently found to have a significant outbreak.^9^ Washington State declared a state of emergency on February 29, large public gatherings were banned on March 11, schools closed on March 13, and a shelter-in-place order was issued on March 23 and is still in place as of April 13. There was a temporal association between the closures and decreased emergency department (ED) patient volumes (Figure 1), now at an average daily census of 73 for the first week of April compared with 175 the same week last year.

**Table 1:** Overview of Response for SCH and CHLA Using the Haddon Matrix

	Physical (ED) Environment	Social (Hospital) Environment	State/County Environment	Agent Environment
	*ED Size*	*Admission Policies*	*First Adult Positive*	*PPE Equipment*
SCH	38 Rooms	Admission process to special infections unit (SIU) developed, rapid admission team developed	Jan 21, 2020 (travel-related)Feb 28, 2020 (first community spread case, WA state)	Early: Gown, double gloves, shoe covers, CAPR, observed donning and doffing Community transmission phase: Gown, gloves, mask with eye protection PPE shortage phase: Extended use CAPR, gown, gloves
CHLA	38 Rooms	Admission process to PUI unit developed	Jan 26, 2020 in LA County (travel-related) Mar 9, 2020 (first community spread case, LA County)	Early: No special PPE, though mask shortage recognized on January 30, 2020 February 18, 2020: N-95 fit testing ramps up Community transmission phase: Gown, gloves, extended use mask with eye protection, N-95 or PAPR for high risk patient or procedure PPE shortage phase: Same as above
	*Negative Pressure Rooms*	*Transfer Policies*	*First Adult Death*	*COVID-19 Testing*
SCH	38 Rooms	No divert and universal accept policy adopted to support regional institutions	February 28, 2020	Jan 2020: DOH testing only with approval (TAT 1-4 days) March 1: Limited testing availability at University of Washington, swabs and universal medium become limited as testing increases (TAT 1-2 days) March 22: SCH test available, swabs limited (TAT 12 hours)
CHLA	3 Rooms	Working to establish policy to support regional hospitals	March 11, 2020	Swabs, reagents, media limited from the beginning ED is testing only patients who get admitted CHLA testing begins March 13, 2020, TAT of 6-14 hours
	*Waiting Room Segregation*	*Resuscitation of PUI policies*	*First Pediatric Positive*	*Staff Illness or Quarantine*
SCH	Typically, 1 waiting room. Hallway repurposed for separate waiting area	Agreement with Anesthesiology to perform intubations on PUIs Tele-resuscitation model developed and simulated with the PICU and Code Blue committees	February 28, 2020	In order of implementation: Work-related travel prohibited Any travel required 14 days quarantine upon return Any known or potential exposure required active temperature and symptoms monitoring Staff testing recommended for any symptoms
CHLA	1 Waiting room and 1 hallway area repurposed to separate symptomatic and asymptomatic patients	Several PUI intubation simulations run through Simulation Center, Decision for no designated PUI intubation service, March 30, 2020, due to low prevalence	March 19, 2020	In order of implementation: Work-related travel prohibited Any travel required 14 days quarantine upon return Staff testing and self-isolation recommended for any symptoms All staff screened at hospital entrance via set of questions (exposure to known positive or any symptoms); if yes, then sent home, arrange testing
	*ED Census*	*Staff Screening or PPE Policies*	*First Pediatric Death*	*EMS Changes*
SCH	Average annual visits, 55 000	Call center set up for staff to call with questions about travel, potential exposure, need for testing	None	Policy for avoidance of field intubation if possible due to exposure risk
CHLA	Average annual visits 95,000	Command Center set up for staff to call with questions about travel or potential exposure	None	Previous guideline already in place that pediatric patients in LA County are not intubated in the field by EMS
	*Alternative Care Sites*	*Hospital-Hospital Agreements*	*City/County/State Quarantine Law*	*Local Closures*
SCH	2 Separate disaster tents set up early in the pandemic response, usual overflow areas not usable	Regional EOC activated Ventilators loaned Decision to accept patients of up to 22 years Regional pediatric patients transferred in to make room at adult hospitals	March 11: Governor limits gatherings March 13: School closures March 23: Stay-at-home order March 25: Non-essential businesses close	All clinics cancel elective and non-urgent visits, transition to telemedicine if possible
CHLA	1 Disaster tent operationalized in parking structure, anticipating surge of patients would be low acuity with respiratory complaints	Active discussion ongoing with regional hospitals and County EMS regarding different levels of surge activation, including possibility of seeing patients of up to 25, 30, and 40 years	March 11: CA governor limits large gatherings March 13: School closures March 19: Stay-at-home order March 19: Non-essential businesses close	All clinics cancel elective and non-urgent visits, elective surgeries canceled, transition to telemedicine if possible ED begins exploring telehealth options

### CHLA: Timeline

On January 26, 2020, the first case of COVID-19 was confirmed in Los Angeles (LA) County in a traveler returning from China. By March 4, there were 7 known cases in LA County and the mayor declared a local state of emergency, with all confirmed cases and close contacts being quarantined. The first case in LA County of community spread was discovered on March 9, and officials began encouraging people to stay at home and avoid large crowds. March 11 marked the first death due to COVID-19 in the county (a 60-year-old female who had a prolonged stay in South Korea). On March 11, the governor limited large gatherings. On March 13, schools in the county closed and, on March 19, there was an official stay-at-home order and closure of all non-essential businesses, which have all remained in effect as of April 13. CHLA also had a marked decrease in census. On March 2, ED daily census was 342 patients; 2 weeks after school closures, the daily census was 185. Patient volumes continue to decline, averaging 80 patients per day in April, compared with an average of 292 patients per day during the same time period in 2019.

### SCH: ED Operations

SCH activated the hospital incident command structure on January 22 in response to the first US case occurring regionally. All branches of incident command were activated (operations, planning, logistics, and finance). The chief medical officer was named the incident commander. Main technical advisors included Emergency Management, Infection Prevention, Workforce and Environmental Safety, and Human Resources. The early activation of the incident command structure allowed for rapid centralized decision-making and flow of information. The initial focus was on readiness of the special isolation unit for the management of a small number of patients and enhanced screening with an evolution into pandemic planning across the organization. Key areas of alignment included screening, personal protective equipment (PPE), isolation, visitation, limiting workforce to essential staff, and testing guidelines. The ED was involved in the initial planning meetings for the organization. The ED operations committee began pandemic preparation on February 12.

Anticipating a possible surge in patient volume, an event tent was procured to be used as additional lobby space after patients were triaged or for evaluating lower-acuity patients depending on clinical needs. For the latter scenario, providers in the tents would wear continuous PPE, including a controlled air purifying respirator (CAPR), gown, and gloves. A multidisciplinary group of nurses and physicians, a pharmacist, and administrative leader worked together to develop the following:

- A provider and nurse schedule of 2-hour shifts (to be taken from ED staffing)
- Patient registration and consent process to occur over the phone
- A paper chart for physician and nurse documentation
- Pre-printed order sheets with common orders
- Pre-printed prescriptions with frequently used medications
- Discharge instructions in our most common 3 patient languages

A second tent was also procured and a city permit was obtained to close a through street, re-route buses, and designate a new ED entrance and screening point if needed (neither have been used to date).

### CHLA: ED Operations

On February 26, the ED leadership team began discussing first steps in preparation for the pandemic. The hospital Manager of Disaster Resource Center and our Director of Infection Prevention and Control were contacted to obtain immediate eye protection for the ED and to start planning for an alternative care site. For planning the latter, we included ED physicians, nurses, technicians, unit assistants, and registration team, as well as hospital stakeholders, including representatives from facilities, security, environmental services, information technology, float pool nursing, CHLA media, the simulation center, and the disaster center.

Based on early reports that the pediatric population was not as critically ill as the adult population, the expectation was that the surge of patients would likely comprise low-acuity patients with respiratory complaints, which could be evaluated in the tent. Trigger criteria were created for use of the tent, with patient inclusion/exclusion screening criteria, a proposed workflow, and all additional equipment necessary (generators, extra electrical, enhanced WiFi, extra computers on wheels, chairs for a waiting area, lighting, extra PPE, and hand sanitizing stations for ED team members as well as patients). On March 13, a drill was performed with all stakeholders present, using mock patients. We determined that no worker could work more than 4 hours in the tent, we created a new electronic medical record (EMR) template for low risk COVID-19 patients, and we obtained access to a COVID-19 discharge instruction template for our EMR system.

### SCH: ED Workflow and Processes

#### Screening and Triage

Screening occurred outside of the ED entrance by RNs and initially focused on international travel. With community spread, it shifted to screening focused on fever or respiratory symptoms for patients or family. If positive, they were masked and placed immediately in a negative pressure room or the symptomatic lobby. As additional COVID-19 symptoms were reported, we adapted our screening to include myalgias, headache, and sore throat. On March 16, we implemented universal staff screening with symptom and temperature checks in accordance with Centers for Disease Control and Prevention (CDC) guidelines.

#### Patient Movement/Cohort

Potentially infectious patients were separated from those at higher risk of infection (ie, oncology patients and neonates). While numbers were low, patients were seen in 1 of 3 rooms that have a back entrance to avoid movement within the ED. Once symptomatic patient volumes increased, patient cohorts were moved to 1 side of our ED. Those who screened positive were masked and brought immediately to a room or placed in the symptomatic lobby with an attempt to keep them at least 6 feet apart, and a second lobby was created for asymptomatic patients. All patient rooms in the ED are negative pressure rooms. The ED has a sliding wall that can be closed in order to further limit air movement between the 2 sides of the ED. This was used during the 2019 Seattle measles outbreak but had not yet been used for COVID-19 patients. The hospital and ED visitor and caregiver policy was updated to allow 1 parent or caregiver to accompany each patient to reduce exposure.

#### PPE Practices and Patient Isolation

The worldwide PPE shortage has become an unprecedented challenge for all hospitals during this pandemic. PPE and patient isolation policies have changed frequently, in response to shortages and updated DOH and CDC recommendations. Initially, we were able to use a high level of protection for any provider in contact with a PUI with a disposable gown, shoe covers, CAPR, double gloves, and observed donning and doffing. After the patient history was obtained by phone, a provider and a nurse entered the room together to examine the patient, perform procedures, and obtain samples as needed.

This process became untenable after community spread. Patients with respiratory symptoms were placed in strict isolation with providers using simple face masks, eye protection, gown, and gloves. If aerosolizing procedures were performed, staff wore CAPRs. Patients at higher risk for COVID-19 due to known exposures or clinical characteristics requiring COVID-19 testing were placed in “PUI isolation” for the duration of their ED stay with a CAPR, gown and gloves for PPE, and observed doffing. CAPR helmets were used due to early severe shortages of N95 masks. N95 use was limited to emergent use when there was no time to don a CAPR. SCH invested in a supply of CAPR helmets during the outbreak of Ebola; with a cart of 22 helmets, we were able to support staff use and even extended their wear. Eventually, the designation between strict isolation due to respiratory symptoms and PUI was no longer relevant, so all symptomatic patients were placed in strict isolation, observed doffing was discontinued, and staff were advised to doff inside the patient rooms, near the door, ideally at least 6 feet from the patient.

By early March, the supply of simple face masks became limited without any additional orders expected. To preserve supply, all masks were moved to a central location. To reduce PPE use, CAPR shields were cleaned and re-used during and across shifts. We emphasized phone communication with patients and families from outside of the room. Staff worked together to limit their frequency of entering rooms, and attending physicians coordinated with trainees and advanced practice practitioners to limit patient exams. For patients and families with negative symptom screenings, standard precautions were recommended. Given increased concern for asymptomatic spread, an extended wear mask policy was adopted for all patients. Clinical staff wore masks outside patient rooms for protection in the clinical space. Due to an inadequate supply, universal masking has not been adopted to date, but we attempt to provide the highest possible level of protection for staff based upon their exposure risk in accordance with the DOH and CDC recommendations. ED spaces were reconfigured to maximize social distancing by closing certain work stations, encouraging 6-foot distancing at staff huddles, and having staff work remotely when appropriate.

#### Testing

Testing capacity has been a challenge for our hospital as it has been across the United States. Initially, testing occurred through the DOH in coordination with the CDC. On March 1, the lab at the University of Washington began processing a limited number of tests with a 24- to 48-hour turnaround time (TAT). They quickly increased capacity, which briefly permitted unrestricted testing. However, due to a swab and universal transport media shortage, testing was prioritized to high-risk patients. Internal testing decreased TAT to a mean of 6 hours. Our testing algorithm followed DOH recommendations and evolved over time to include severe respiratory illness requiring admission, patients with respiratory illness and significant comorbidities, and those with known COVID-19 exposures. More recently, we have started testing all patients being admitted and/or undergoing operative procedures to identify the COVID-19 status of patients requiring aerosolizing procedures and to preserve PPE for those who are negative. As of April 13, we have tested 1655 patients and have 25 positive results (1.5%). On March 12, we launched drive-through testing for employees with symptoms of COVID-19. As of April 13, we have tested 1065 members of the workforce with 40 positive results (3.8%).

### CHLA: ED Workflow and Processes

CHLA ED typically has 1 screener nurse who identifies the chief complaint and then places the patient in a room based on acuity category, or to a waiting area if all ED beds are full. In early March, we began travel screening. By mid-March, with community spread, this was no longer relevant, though we did begin asking patients or their caregivers whether they had a known COVID-19 contact. In addition, we enforced a 1-caregiver-per-patient policy to reduce the potential exposure to our staff.

On March 13, CHLA began in-house COVID-19 testing. Depending on the time that the sample reaches the laboratory, the TAT ranges between 6 and 14 hours. As of April 12, we have sent 1299 tests throughout the organization and have had 11 positives (0.8%). In addition, we have tested 723 staff members with 33 positive results (4.6%).

On March 19, the hospital opened an inpatient PUI unit. It was decided that all admissions from the ED would have COVID-19 testing. Symptomatic patients with respiratory symptoms would be admitted to the PUI unit prior to test results. Asymptomatic patients requiring admission would also be tested, but, in an effort to conserve PPE, would wait in the ED until COVID-19 results were completed, which has led to lengths of stay between 7 and 20 hours. All non-emergent procedures (operative and interventional radiology) also require COVID-19 testing results prior to the procedure to conserve PPE and equipment for negative patients. Though our microbiology laboratory has actively been ramping up testing and decreasing TAT, requiring test results for this large group of patients has resulted in significant delays, depending on the time that the COVID-19 test was sent. However, given the low census and the overall needs of the hospital, the ED has continued this protocol.

On April 2, we changed our usual workflow and separated our symptomatic from asymptomatic patients in the waiting room and in the ED with the goal of minimizing possible cross contamination and spread. We developed an asymptomatic waiting area with the shortest path of travel to a single asymptomatic ED zone. We designated our large main waiting area and 3 ED zones for symptomatic patients. However, we soon noted the decline in symptomatic patients seeking emergency care. Therefore, on April 6, we changed our zones to have 3 asymptomatic zones and 1 symptomatic zone.

### CHLA: Aerosolized Medications and Procedures

Approximately 1 month prior to the pandemic, our ED had initiated a new protocol to use albuterol metered-dose inhalers (MDIs) for patients ≥ 2 years of age presenting with a mild asthma exacerbation. Patients who qualify are given an albuterol MDI with the hope of educating our families on proper use as well as giving them a short supply of the medication. With the pandemic, we initially thought to expand the inclusion criteria for asthmatics to receive albuterol via MDI to decrease exposure risk during the aerosolized medication given the lack of N95 masks, but we were quickly faced with a nationwide albuterol MDI shortage. Because of the critical shortage of PPE, we began using powered air purifying respirators (PAPRs) for all aerosolized procedures, including albuterol nebulization, advanced ventilatory support, and intubation.

### SCH: Resuscitations

Given the limited history when a high-acuity patient arrives to the ED, we determined that all patients requiring immediate resuscitation would be considered a PUI until more information was available. A small supply of N95 masks was placed by the resuscitation room for emergent use. As staff adopted extended CAPR use, staff already wearing CAPRs responded first to resuscitations so that they can enter the room faster. Iterative simulation was employed to design, test, and orient staff to a modified resuscitation team structure using telehealth equipment. We placed a tablet inside of the room and computer outside of the room to create a 2-way video and audio connection to decrease the number of staff physically in the room, to reduce PPE use, and to limit staff exposures during resuscitations. This decreased the number of staff in the room for resuscitations by approximately 50%–70%. Additional simulations refined the COVID-19 intubation process. We have also been working with Anesthesiology to standardize the process of intubation for a PUI, using extra protective drapes and video laryngoscopy.

### CHLA: Resuscitations

Simulation teams began “COVIDtubation” in situ simulations in the ED and intensive care units on March 16. All patients requiring immediate resuscitation would be considered a PUI, including trauma patients, and would be directed to the resuscitation rooms within the newly formed symptomatic zone. The preferred PPE is PAPRs for resuscitation, and after 1 cardiac arrest, during which communication through PAPRs was difficult, the hospital provided a 5-way communication radio available underneath the PAPRs for clearer communication. Further simulations tested the optimization strategy and a radio-based communication paradigm. Subsequent resuscitations have had mixed adherence to PPE recommendations, and the cultural shift from a large group resuscitation to limited personnel and closed doors remains a challenge. External consultants like Anesthesiology are available but are not automatically called for PUIs. Further work is ongoing to develop protective barriers, such as acrylic boxes and plastic drapes during intubation, with the collaboration of Anesthesiology colleagues.

### SCH: Staff Communication

As the pandemic unfolds, practices and policies in our hospital evolved rapidly. Given the large number of staff rotating on a daily basis, keeping everyone up to date has been challenging. Initially, e-mail communications and twice daily staff huddles were the primary methods for dissemination, but staff quickly expressed being overwhelmed at the amount of e-mails they received. We have since developed an ED operations committee specific to COVID-19 that does rounds in person in the ED to share updates and receive feedback. We also send 1 daily e-mail update with a summary of new information. The hospital developed a central Intranet page dedicated to COVID-19 practices and updates.

### CHLA: Staff Communication

While the pandemic was spreading, the hospital Command Center began 4 times per day meetings, which were quickly decreased to twice a day for the less critical stakeholders. On March 18, the ED initiated twice daily remote meetings for the ED provider team to discuss issues and keep everyone updated as the situation was so fluid in the early days of the pandemic. After 1 week, we changed the remote meeting to once daily, and, after 2 additional weeks, we transitioned to 3 times per week for these remote updates. These meetings have been highly attended, whether remotely via video or by phone. We often have more than 30 physicians and nurse practitioners on the line at any given time.

### SCH: Future Planning and Regional Response

Given that we are in the early stages of this pandemic and models still suggest potential for a large patient surge and the possibility of a biphasic pandemic, planning is ongoing for our hospital and region. We are working to share equipment and supplies when possible. Our hospital has increased the upper age to which we accept patients to 22 years and are making plans to increase this further if needed. Simultaneously, we are creating aids for staff who do not frequently care for adult patients. We have accepted inpatient transfers of pediatric patients from community hospitals to increase their capacity for adult patients. In the ED, we have decreased staffing to preserve capacity for the possibility of subsequent patient surges or coverage of ill staff. We continue to explore roles that can be done remotely, including medical control calls or consultations with community physicians.

### CHLA: Future Planning and Regional Response

Although it is unclear whether we will experience a surge of pediatric patients, we are continuing to prepare. We have decreased our nurse and physician staffing hours significantly while our census remains low, to preserve the ability to up staff if needed during a surge. The hospital is discussing training nurses who currently do not work in a patient care capacity to work back at the bedside if needed. We are working under the Children’s Hospital Association surge contingency planning on accepting all pediatric patients from regional hospitals to free up space for adult patients at those centers. We are also discussing the steps needed to accept adult patients up to 40 years old, depending on the surge level and needs of the region, and possibly having access to consultation with our partners at Keck School of Medicine of the University of Southern California.

## CONCLUSION

The onset of the global COVID-19 pandemic has demonstrated the need for hospital and ED operations to be mobilized rapidly and to remain flexible and effective. It has been critical for both hospital and ED leadership to be proactive in anticipating needs, formulating guidelines, and executing new operations. The ED staff members have needed to show tremendous flexibility and resilience in order to adapt to the ever-changing workflow and guidelines. The sheer amount of information and detail needed to be discussed with staff members has been staggering, and finding the correct balance of 2-way communication via e-mails and remote meetings has been critical. It has also highlighted the importance of a coordinated regional response as institutions are affected differently and crucial items remain in short supply.
